# Diagnosis and Treatment of Borderline Lesions

**DOI:** 10.1080/135771402753667646

**Published:** 2002-05


### DIAGNOSIS AND TREATMENT OF BORDERLINE LESIONS

### ORAL PRESENTATIONS

### A01 Desmoid Tumours. A Clinical Review of 30 Patients with more than 20 Years Follow-up

B.P.M. Dalén[1], P. Bergh[2], K. Berlin[3], B. Gunterberg[2]
*[1]Sahlgrenska University Hospital, Goteborg, Sweden, [2]Sahlgren University Hospital, Goteborg, Sweden, [3]University of Goteborg, Goteborg, Sweden*

*Objective:* To assess the long-term outcome in patients with desmoid tumours.
*Methods:* Thirty consecutive patients (20F, 10M) with a minimum follow-up of 20 years (mean 28 years) were reviewed. The tumours were located in the abdominal wall (7), upper extremity (6), lower extremity (6), back (5), thoracic wall (4), head-neck region (1). One patient had multifocal tumours. Twenty-nine patients had surgical treatment. Three received radiotherapy later in the course of the disease. One had anti-estrogenic treatment in addition. The surgical margins were wide/radical (10), marginal (13) or intralesional (6). One patient had radiotherapy alone.
*Results:* At follow-up fifteen patients were continuously disease-free after the first surgical treatment, nine patients had no evidence of disease after surgery for recurrent tumours. Five patients were alive with stable tumours. One tumour disappeared gradually after radiotherapy only (39 Gy). Two patients died of non-tumour-related causes 20 and 23 years after diagnosis. The overall recurrence rate was 40%. Only 1 patient with wide/radical margin had a recurrence. The mean time to first recurrence was 1.5 years. Two patients were amputated, 4 patients had moderate – severe post-treatment symptoms.
*Conclusion:* Desmoid tumours have an unpredictable development. Long-term survival is the rule regardless of recurrences. Some disabling symptoms may be more related to over-treatment than the tumour itself. New strategies are needed and are under way.

### A02 Diagnostic Impact of Histologic Parameters in Differentiating Enchondroma from Grade I Central Chondrosarcoma

D. Eefting[1], M.J.A. Geirnaerdt[2], S. Le Cessie[3], A.H.M. Taminiau[4], P.C.W. Hogendoorn[1]
*Departments of [1]Pathology, [2]Radiology, [3]Medical Statistics and [4]Orthopedic Surgery, Leiden University Medical Center, Leiden, The Netherlands*

*Introduction and objective:* Because of the difference in clinical management of enchondromas and central grade I chondrosarcomas it is important to make a correct preoperative diagnosis. In this study we investigated the diagnostic value of histologic parameters in differentiating these tumours.
*Materials and Methods:* Cytologic and tissue-architectural features of 57 patients (20 enchondromas and 37 central grade I chondrosarcomas) were assessed. The observer was aware of the tumour localisation, age and clinical symptoms (swelling, pain or fracture). A final diagnosis, using at least 10 years of follow-up after the biopsy in case of diagnosis of enchondroma was assessed in a multidisciplinary setting with full clinicoradiological and pathology data available. Tumours of the digits and toes were excluded. The chi-square test, classification trees and logistic regression were used to assess the discriminating power of individual parameters and to find an optimal set of differentiating parameters.
*Results:* Entrapment of host lamellar bone, high cellularity, marked nuclear pleomorphism and irregular cell distribution showed the greatest discriminating value ($p<0.001$). The combination of high cellularity, presence of host bone entrapment, open chromatin, mucoid matrix quality >= 20% and age above 45 years allowed a perfect prediction of diagnosis. A classification tree illustrated that if mucoid matrix degeneration is more then 20% or host bone entrapment is present (or both), the tumour is with close to certainty malignant.
*Conclusion:* Combination of 5 parameters (high cellularity, presence of host bone entrapment, open chromatin, mucoid matrix quality >= 20% and an age above 45 years) allowed optimal differentiation. With a simple classification tree, based on 2 parameters (mucoid matrix degeneration more then 20% or host bone entrapment present or both), 54 of the 57 (94.7%) cases were assessed correct (sensitivity 95% and specificity 95%). This approach, combined with tailored radiology, will lead to optimal classification.

### A03 Bcl-2 Immunohistochemistry as a Tool to Distinguish Osteochondroma and Grade I Peripheral Chondrosarcoma

J.V.M.G. Bovee, P. Kok, L.J.C.M. Van den Broek, P.C.W. Hogendoorn
*Leiden University Medical Center, Leiden, The Netherlands*

*Objective of study:* Peripheral chondrosarcoma arises within the cartilaginous cap of an osteochondroma (osteocartilaginous exostoses). Osteochondromas occur solitary, or within the context of hereditary multiple exostoses (HME). To distinguish between benign and malignant can be difficult, both clinicoradiologically as well as histologically. Recently we documented an upregulation of PTHrP and Bcl-2, two proteins presumed to act downstream of EXT (the genes involved in HME), characterising the progression of osteochondroma towards peripheral chondrosarcoma. Our goal was to evaluate whether the immunohistochemical detection of Bcl-2 and/or PTHrP can be used in the differential diagnosis between osteochondroma and grade I peripheral chondrosarcoma.
*Methods:* Using the national pathology database (PALGA), we retrieved tissue blocks of 29 laboratories in The Netherlands, including 73 osteochondroma specimens of 57 patients and 43 grade I chondrosarcoma specimens with documentation of a (previous) osteochondroma of 40 patients. Two bone tumour pathologists reviewed diagnoses. PTHrP and Bcl-2 immunohistochemistry was performed and scored semi-quantitatively by two independent observers.
*Results:* Bcl-2 expression was absent in osteochondromas (1/56 positive), while an upregulation was observed in a subset of chondrosarcomas (18/37 positive) ($p=0.000$ Fisher's Exact Test). Bcl-2 immunohistochemistry proved to be a strong diagnostic tool. In case of any staining, both specificity and positive predictive value were 59%. In case of moderate to strong or more diffuse staining, a high specificity of 98% was found with a positive predictive value of 95%. Sensitivity was however relatively low (89% and 49% respectively). For PTHrP, variably intense staining was found in a

ISSN 1357–714X print/1369–1643 online/02/S1/S7–49 © 2002 Taylor & Francis Ltd varying percentage of cells in both osteochondromas and chondrosarcomas, which made it a less suitable diagnostic marker.
*Conclusion:* Bcl-2 Immunohistochemistry is a valuable immunohistochemical test to distinguish osteochondroma and grade I peripheral chondrosarcoma, in which moderate to strong or diffuse cytoplasmic staining is highly suggestive (95%) of malignancy.

**A04 The Treatment of Benign and Low-grade Malignant Intramedullary Chondroid Tumours with Curettage and Cryosurgery**

H.W.B. Schreuder
*University Medical Centre St. Radboud, Nijmegen, The Netherlands*

*Introduction:* Much controversy exists about the methods of staging and treatment of enchondroma and low-grade chondrosarcoma, because accurate distinction is hampered by their radiographically and histologically similarity. The continuous spectrum of biological behaviour of these entities is unpredictable and a benign lesion as enchondroma can even differentiate in secondary chondrosarcoma. Because of low recurrence risk limited surgery with or without adjuvant therapy is advocated.
*Method and patients:* Since 1991 we treated 97 intramedullary chondroid lesions in 94 patients with intralesional resection, local adjuvant cryosurgery and bone grafting. All cryosurgical procedures were per-operatively monitored using thermosensors and real-time computerised graphical visualisation. Sixty-five patients with 68 lesions had a follow-up of 2 years or more and are therefore included in this study. All patients had a standard follow-up consisting of local radiographs and bone scan. MRI, CT and chest X-ray were done on indication.
*Results:* One lesion (an aggressive enchondroma located in the hand) recurred. No metastases were observed. Complications included one deep wound infection and 7 fractures; none of these patients underwent preventative osteosynthesis. All bone grafts incorporated, resulting in full-weight-bearing capacity and good or excellent functional results.
*Conclusion:* For enchondroma (stage 2 and 3) and chondrosarcoma grade 1 (stage IA), this study showed that the combination of curettage and cryosurgery as adjuvant therapy can be considered to be equal as marginal resection in terms of local tumour control. In contrast to marginal resection the treatment described will not leave segmental bone defects, which are difficult to reconstruct. Therefore all patients had good to excellent functional results. Fracture is of concern, but along the learning curve and since the use of preventative osteosynthesis this complication was not encountered anymore.

**A05 The Atypical Lipoma**

S.M.M. Sommerville, J.T. Patton, J.C. Luscombe, R.J. Grimer
*Royal Orthopaedic Hospital, Birmingham, United Kingdom*

*Aim:* Significant controversy exists with regard to the nomenclature, treatment and outcome of a group of well-differentiated lipomatous tumours sometimes labelled as atypical lipomas. The purpose of this paper is to attempt to clarify these controversies by reporting our experiences with this lesion.
*Methods:* Seventy-five patients with the diagnosis atypical lipoma and a minimum two-year follow-up were identified from the oncology service database at our institution. Records were reviewed to record the oncological outcome with particular note of any local recurrence, metastasis, or dedifferentiation.
*Results:* Treatment of atypical lipomas at this institution is by marginal resection alone. Sixty-five patients were treated here with their primary lesion. Follow-up was a minimum of two years with a mean of 49 months (range 24 to 114 months). Five patients had a local recurrence (7.7%). Four recurred once and one recurred twice. No patient had a metastasis or died as a result of the tumour. No lesion dedifferentiated. Nine of the ten patients seen here with a presumed recurrence actually had a recurrent atypical lipoma. Recurrences were treated by further marginal resections and one went on to have a further recurrence. None of these patients had a metastasis and no lesion dedifferentiated. The final patient with a suspected recurrence most likely had a radiation-induced sarcoma nine years following radiotherapy after the marginal excision of a recurrent atypical lipoma.
*Conclusion:* We believe the term atypical lipoma is appropriate for these tumours, as they appear not to have any metastatic potential, merely a propensity to recur locally. The chance of dedifferentiation is very small and the role of radiotherapy in the causation of dedifferentiation is uncertain. We suggest that a simple marginal resection (shelling-out) is adequate treatment for these lesions. Radiotherapy should not be used.

### POSTER PRESENTATIONS

**A06 IHH/PTHRP and BMP Receptors Expression Discriminate among Cartilaginous Lesions**

J. Warzecha, E. Lucarelli, L. Zanella, M. Alberghini, G.A. Gobbi, V. Maini, P. Bacchini, P. Picci, F. Bertoni, L. Sangiorgi
*Istituti Ortopedici Rizzoli, Bologna, Italy*

*Objective:* Indian Hedgehog (and its downstream target) and Bone Morphogenic Proteins have shown to play a relevant and independent role in chondrocyte proliferation and regulations during embryonic development. Aim of this study is to evaluate if the expression of these molecules can be used for diagnostic classification of cartilaginous lesions.
*Methods:* 92 cartilaginous lesions were analysed by immunohistochemistry with the antibodies for Indian Hedgehog (Ihh), Parathyroid Hormone related peptide (PTHrp), Bone Morphogenic Protein Receptors type 1 A (BMPR-IA), type 1B (BMPR-IB) and type 2 (BMPR-II) receptors. Staining was evaluated by 4 investigators independently.
*Results:* We observed a distinct pattern of expression of these proteins in specific subset of cartilaginous lesions. Ihh and BMPR-II expression were found in 4,9% and 5,1% of osteochondromas. In contrast, the same antigens were expressed in 54,1% and 48,3% of peripheral chondrosarcomas. In central chondroid lesions, two different proteins, PTHrp and BMPR-IB showed a similar pattern of expression distinguishing between enchondromas (11,1% and 25%) and grade I chondrosarcomas (67,7% and 79,5%).
*Conclusion:* Immunohistochemical detection of these proteins could identify different subset of cartilaginous lesions and could be useful for diagnostic purposes.

**A07 Impact of Limited Resection on Outcome in Patients with (OFD-like) Adamantinoma**

H.E. Henkus[1], B.H.W.G.M Smit[2], H.M. Hazelbag[3], P.C.W. Hogendoorn[1], A.H.M. Taminiau[1]
*[1]Leiden University Medical Center, Leiden, The Netherlands, [2]Department of Orthopaedics, Vancouver, Canada, [3]Department of Pathology, Leiden University Medical Centre, Leiden, The Netherlands*

*Background:* (osteofibrous-dysplasia-like) adamantinoma is an intracortical lesion with various degrees of intramedullary involvement. Intercalary grafting with osteosynthesis or amputation is associated with significant morbidity. Using MRI a more sparing procedure might be considered that preserves the continuity of the bone. Reconstruction with inlay graft and minimal osteosynthesis allows future MRI.

*Objective:* Evaluate middle/long-term functional and oncologic Results of en-bloc resection with reconstruction with auto-or allo inlay grafts with minimal osteosynthesis in patients with (OFD-like) adamantinoma of the long bones.

*Methods:* Clinical and radiographic data of 18 patients between 1986 and 2001 were reviewed. The diagnosis (OFD-like) adamantinoma was based on radiographic and histopathological findings.
Sex: male: 6; female: 12
Symptoms none: 3 pain/swelling: 15
Location: diaphysis tibia: 16 metaphysis tibia: 2
Diagnostic procedure: jamshidi biopsy: 12 incisional biopsy: 3 curretage: 2
Histological classification: OFD-like adamantinoma: 13 classic adamantinoma: 4
Treatment: inlay graft: 13 Intercalary graft preserving a periosteal sleeve: 5
Average age at the time of surgery: 23 years (8–38 years). Surgery: MRI-guided en-bloc resection. Reconstruction: tibial/fibular auto/allograft. Osteosynthesis: no to 4 screws. Follow-up: repetitive X-rays and MRI. Incorporation was defined as the absence of a radiolucent line at the host-donor junction with smooth external continuity of cortical bone. Functional outcome: MSTS-score.

*Results:* The average length of defect: 11.3 cm (6–21 cm). Complications: Intra-operative: fracture host cortex: 2 (both needed osteosynthesis, one after casting because of hypertrophic pseudoarthrosis). Postoperative: infections/wound problems: 0, neurological: 1 (dropfoot), reflex dystrophy: 1. Late complications: fracture through graft: 2 (both because of trauma) Average immobilisation-time: 3 months. Average graft consolidation-time: 8 months (2–24). Mean disease-free interval: 69 months (1–149). Local recurrence: 1 (with sarcomatous dedifferentiation. Died of metastatic disease). Average MSTS-score: 24/30 [80% (range 0–100)].

*Conclusion:* En-bloc resection with reconstruction using an inlay graft with minimal osteosynthesis is an oncologically sound procedure with good functional result. The risk of a fracture should be minimised by using cast or brace protection sometimes for a prolonged period.

### A08 Enchondroma – Indications for Surgical Treatment and Follow-up

P.E.M. Mueller[1], H.R. Dürr[1], B. Wegener[1], A. Von Liebe[1], C. Pellengahr[2], V. Jansson[1]
*[1]University of Rostock, Germany, Rostock, Germany, [2]University Munich Grosshadern, Munich, Germany*

Enchondromas are benign cartilaginous tumours. Rarely these transform into chondrosarcomas. Curettage and bone grafting is usually performed assuming a low rate of complications. We analysed data from 73 patients with enchondromas treated by curettage between 1980 and 1997 retrospectively with respect to symptoms, therapy, complications and recurrences. For comparison we included data from all cases of chondrosarcomas in the same period. 68 patients had solitary and five multiple enchondromas. All patients were treated by curettage of the tumour, in most cases followed by cancellous bone grafting. 23% of patients suffered a complication. There were two recurrences but no malignant transformation in the follow-up period. In the same time period 29 patients with chondrosarcomas were treated, including two secondary chondrosarcomas. One had a recurrence of a benign enchondroma of the fifth metacarpal (no histological signs of malignancy) and developed a pulmonary metastasis of a chondrosarcoma 52 months later. The other is a patient with Ollier's disease with several enchondromas at different sites who had a secondary chondrosarcoma of the right radius.

*Conclusion:* Malignant transformation of a solitary enchondroma to a chondrosarcoma is rare. But the complication rate of enchondroma curettage is considerable. Thus regular follow-up of asymptomatic enchondromas with radiographs may be the better option.

### A09 Differentiation of Enchondroma and Low-grade Chondrosarcoma – Clinicopathological and Radiological Findings in 34 Cases

H. Welkerling[1], S. Kratz[2], G. Delling[3], V. Ewerbeck[4]
*[1]Universitaetsklinik fuer Orthopaedie, Graz, Austria, [2]Inst. Of Diagnostic Radiology, Erlangen-Nuernberg, Germany, [3]Hamburg University Institute of Pathology, Hamburg, Germany, [4]Depart. Of Orthopaedic Surgery, Heidelberg, Germany*

18 low-grade chondrosarcomas (CS 1) and 16 enchondromas (EC) were analysed in a retrospective study. The purpose of the study was to compare histological, clinical and radiological finding of EC and CS 1 in order to select criteria of malignancy.

*Methods:* 18 patients with CS 1 and 16 with EC were treated at the Department of Orthopaedic Surgery of Heidelberg University. The study included analysis of histological material, evaluation of x-rays and clinical data. EC were strictly differentiated from CS 1 by growth pattern.

*Results:* The mean age of patients with EC and CS 1 was similar (44 versus 46 years). The mean follow-up time was 40 months in CS 1 and 29 months in EC. 87.5% of CS 1 patients reported pain, whereas only 43.8% of EC did. This difference was significant (p=0.009). Cellularity, the quantity of double nuclei and mitosis did not show any significant differences between EC and CS 1. Both entities are characterised by small, isomorphic nuclei. Significant difference could be observed concerning cortical destruction between EC and CS 1 (19% versus 94%). None of the EC, but 44% of the CS 1 lesions had an extra-osseous component. Local recurrence could not be observed in EC, but in 11% of CS 1 after intralesional or marginal procedure.

*Conclusion:* In contrast to patients with EC, those with CS 1 usually had history of pain, and the radiological findings of CS 1 were much more aggressive. EC and CS 1 are distinguishable by growth pattern, but not by cytological features. Cortical destruction, the presence of an extra-osseous component and pain are suspicious of malignancy in differentiation of EC and CS 1, especially if the histological findings are unclear.

### A10 Surgical Treatment of Border Line and Low Grade Malignant Intramedullary Chondroid Tumours

P.D. Daolio, G. Oldani, G. Perrucchini, F. Lazzaro, R. Zorzi, P. Zacconi, A. Parafioriti, S. Mapelli
*Istituto Ortopedico 'Gaetano Pini', Milano, Italy*

Some chondroid lesions of bone represent a considerable problem for the pathologist because the distinction between benign and low-grade malignant tumours should be hard to share. We revised the patient files in all cases with cartilaginous lesions classified as borderline or low-grade chondrosarcoma (stage I A). From 1957 to 1988 we treated 84 patients with chondroid tumours. 24 of them were classified as low grade or borderline lesions. Most of them were treated by bone resection. Since 1988, according to other Authors studies, low-grade malignant and borderline lesions of long bones, have been treated by deep curettage, adjuvant and acrylic cement. From December 1988 to February 2001, 114 patients were operated because of a cartilaginous lesion. 39 were classified as low grade or borderline lesions (27 intramedullary and 12 peripheral lesions). We studied the central lesion: in 18 patients we performed deep curettage, adjuvant and acrylic cement, 9 patients were treated by bone resection (pelvic and spine sites). Although the follow up of 7 of them, is less then 5 years, as all the patients are continuously disease free, conservative surgery seems to be a satisfactory method for local control of these tumours.

**A11 How Spreads GCT?**

M.S.J. Mikel, E.N. 1 Noain, C. Villas
*University Clinic of Navarra, Pamplona, Spain*

*Objective:* Bone GCT is supposed to be a benign lesion, but it can produce pulmonary metastases. Some times local control can be achieved by intralesional surgical procedures, such as curettage, but en-block excision is preferable for avoiding recurrences. The authors present a series of GCT treated in the same institution focusing in the ways of spreading of those which recurred.
*Methods:* Since 1967, 57 GCT were treated at our institution and 16 were referred after being treated elsewhere. Expert pathologists made histological diagnosis and grading according to Jaffe scale. The most frequent locations were distal femur and proximal tibia. Simple x-rays of the location of the tumour and chest were used for the follow-up in all cases. MRI was used also since 1990.
*Results:* 17 cases recurred in the primitive bone location, two in an adjacent bone, one in a distant bone, 1 in the soft tissues surrounding the bone, and one in distant soft tissues. Two cases progressed to fibrosarcoma after irradiation elsewhere. No pulmonary metastases were seen in this series, except in the aforementioned cases of fibrosarcoma. No cases of lymph node dissemination were seen. No relationship between histological grade (Jaffe) and recurrence was seen.
*Discussion:* GCT can not only locally recur, but also can spread to the surrounding bones and soft tissues. A case of intra-arterial spread to distant soft tissues was also seen. The mechanisms of spreading of this tumour remain controversial, since even after en-block excision recurrence is possible, while other cases may be cured after curettage. The use of adjuvants such as cement, phenol or cryosurgery can diminish the risk of recurrence but no data were found to predict the outcome.

**A12 Intra-articular Osteoid Osteomas**

P. Cool, V. Pullicino, D. Williams, A. Darby
*RJAH Orthopaedic Hospital, Oswestry, United Kingdom*

It has been reported that osteoid osteomas regress spontaneously and that treatment can be conservative with anti-inflammatory medication. Others advocate active treatment with thermocoagulation of these lesions. Intra-articular Osteoid Osteomas do normally not have the intense reactive bone formation as is often seen in Osteoid Osteomas in other locations. Consequently, juxta-articular Osteoid Osteomas are more difficult to diagnose and the diagnosis is often made late. We investigated the natural history of intra-articular Osteoid Osteomas that presented to our centre. We also reviewed 10 patients with intra-articular Osteoid Osteomas that had been reported by our centre 10 years previously. The diagnosis was histologically proven in all cases. All patients reviewed had early radiographic arthritic changes to the involved joint and one patient required joint replacement surgery for end stage osteoarthritis. It is postulated that Osteoid Osteomas originate an intense inflammatory reaction whatever there location. If the location is juxta-articular, it results in intense synovitis rather then the more classical periostitis with reactive new bone formation. We recommend that once a diagnosis of intra-articular osteoid osteoma is made, the patient should be treated actively with thermocoagulation. Delay in treatment will result in early arthritis with destruction of the joint.

**A13 Treatment and Outcome of Low-grade Chondrosarcoma**

D.A. Campanacci, G. Beltrami, P. De Biase, A. Astone, M. Pietri, R. Capanna
*Centro Traumatologico Ortopedico, Firenze, Italy*

Low-grade chondrosarcoma (CHS) (grade 1) has a scarce tendency to local recurrence and metastatic dissemination. Some cartilaginous lesions present clinical, radiographical and histological features, which are, defined borderline between a benign chondroma and a malignant CHS. The appropriate approach towards these lesions has not been clearly defined yet and surgeons are debating between clinical observation of the patient and surgical treatment with curettage or resection. The authors analyse their experience on low-grade CHS with the aim to define surgical guidelines on the basis of clinical, radiographical and histological considerations. A series of 32 grade 1 CHS was reviewed occurring in 31 patients (one patient had two localisations). Seven patients had a peripheral CHS and in 25 cases a central CHS was observed. The surgical treatment was a resection in 26 cases and intralesional curettage in 6 cases. At an average follow-up of 44 months, no local recurrence and/or metastatic dissemination was observed in 30 cases, while in two patients, both treated with surgical resection, a local recurrence occurred. Both patients underwent an amputation and a progression of malignancy to a high-grade CHS was observed at histology. In both cases, metastatic dissemination occurred. From the analysis of the data it appears that a lesion showing the radiographical features of a cartilaginous tumour, when it is painless, with no endosteal cortical scallopping, extending for less than 5 cm in the medullary canal, should be followed in time with clinical and radiographical observation. In the other cases, a biopsy should be performed of a specimen including the cortex and the transition area between the cortex and the tumour. When the histological diagnosis indicates a borderline lesion an accurate curettage with the use of local adjuvants is recommended. In case of grade 1 CHS, a resection with wide margins should be performed.

**A14 Medial Acetabular Cysts – A Cause for Concern**

J. Rees, S.O. Hill, D.H. Williams, V. Pullicino, P. Cool
*The Orthopaedic Hospital, Shropshire, United Kingdom*

*Introduction:* Acetabular cysts are a common finding in patients with osteoarthritis of the hip. These mainly occur in the weight bearing superior part of the acetabulum. At our institute we have had 7 cases of medial acetabular wall cysts, which presented with hip pain mimicking that of osteoarthritis. All these cysts turned out to be malignant in origin. The aim of this study was to identify the distribution, position and multiplicity of acetabular cysts in osteoarthritis of the hips and highlight the importance of the finding of medial acetabular cysts.
*Methods:* The radiographs of the 7 cases of medial acetabular cysts were analysed and compared with the pre-operative radiographs of 100 consecutive patients who had a total hip replacement for osteoarthritis. The presence of acetabular cysts was recorded, as well as their number and location within the acetabulum, using a modification of DeLee and Charnley zones. All patients had a minimum follow-up of 1 year. We excluded patients with any history of malignant disease or inflammatory arthropathy.

*Results:* Overall half the cases of osteoarthritis were associated with acetabular cysts. All had other radiographic features consistent with osteoarthritis. The majorities of the cysts were multiple in number and were found in the weight-bearing supra-medial and supra-lateral region of the acetabulum. None were found to involve the medial wall of the acetabulum. Of the malignant medial wall cysts, none had features suggestive of osteoarthritis.
*Conclusion:* Multiple and solitary acetabular cysts are common in patients with osteoarthrosis. They occur predominately in the weight bearing zones of the acetabulum and are always associated with other osteoarthritic changes. Isolated medial acetabular cysts do not occur in osteoarthrosis and require further investigation to confirm their nature.

**A15 Is Treatment with Curettage and Phenol Allowed for Low Grade Central Chondrosarcoma?**

S.H.M. Verdegaal, P.C.W. Hogendoorn, A.H.M. Taminiau
*Leiden University Medical Center, Leiden, The Netherlands*

*Introduction:* Low-grade central chondrosarcomas of the extremities were treated by local resections with wide margins. Although it is adequate surgery, morbidity is to concern. Since 1994 we started to treat low-grade chondrosarcoma by intralesional surgery (curettage) in combination with adjuvant therapy (phenol).
*Purpose:* Is treatment with curettage and phenol safe and allowed for low-grade chondrosarcoma?
*Methods:* From 1988 on 23 patients were treated for low-grade chondrosarcoma. (10 resection, 13 curettage/phenol). PA-diagnosis: 7 borderline, 16 grade I chondrosarcoma. Excluded are low-grade chondrosarcoma of juxtacortical origin, or secondary to osteochondroma. All cases were controlled by X-rays and repeated dynamic MRI.
*Results:* Resection group (10), female (7), male (3), average follow up 93,6 months (42–164), Proximal femur (3), distal femur (2), proximal humerus (1), distal humerus (1), proximal fibula (1), radius (1) and digits III (1). For reconstruction allograft (3), allograft and prosthesis (4) or prosthesis (1) were used. Mean surgery time 3 h 30 min, average admission 13.6 days (4–28).
*Complications:* 2 non-union allograft (28.5%), 1 subcutaneous infection. Intralesional group (13), female (9), male (4), average follow up 62 months (45–89). Distal femur (6), proximal tibia (1), proximal humerus (4), metacarpal bone (1). Mean surgery time 1 h 25 min, average admission 4.6 days (3–10). 12 Patients were treated by adjuvant phenolization after curettage. 1 Male was treated with curettage alone, because cortex was destructed. This patient had the only recurrence, after 2 years. In no other case, controlled by X-ray or MRI, recurrence was found.
*Conclusion:* Although we realise that follow up time is still short and number of treated patients is relatively small, only one recurrence is seen (curettage without phenol). No other recurrence or metastasis is seen and all patients are still alive. In our opinions there is a place for intralesional treatment of central borderline and grade I chondrosarcoma, followed by adjuvant therapy. Longer follow up is necessary, because local recurrence might be expected in the intralesional surgery group.

**A16 Clear Cell Chondrosarcoma (CCC): The Rizzoli Institute Experience**

M. Donati, A. Colozza, L. Perna, P. Picci, F. Bertoni, M. Mercuri
*Istituti Ortopedici Rizzoli, Bologna, Italy*

From 1969, 24 cases of clear cell chondrosarcoma (CCC) were registered in the Rizzoli Bone Tumour archive. The histological review excluded 5 more cases: 4 due to more aggressive attitude (2 dedifferentiation in Osteosarcoma and 2 high grade Chondrosarcoma) and 1 resulted as a Chondroblastoma. In the left 19 cases, age ranged from 24 to 61 years (mean 37), tumour was located in the proximal femur in 9 cases (47%), proximal humerus 2, acetabulum 2, and distal femur, proximal tibia, scapula, 1st lumbar vertebra, 5th rib and talus 1 each. Eight cases were previously treated in other institutions (7 curettages and 1 osteosynthesis), while our treatment was resection. Among the 11 cases primarily treated by us, 3 were curetted. Pain was present at diagnosis from 3 to 48 months (mean 24). In 5 cases the patient arrived to the hospital because a pathologic fracture. Fourteen patients have been followed more than 5 years: time ranging from 74 to 241 (mean 140) months. The classical roentgenographic feature of a CCC is a clear margin, lytic lesion in the proximal femoral neck. It is frequently misinterpreted as a chondroblastoma, central chondrosarcoma or giant cell tumour; however, in other locations the tumour may mimic several more lesions as osteoblastoma, low grade osteosarcoma and fibrosarcoma. Only two patients experienced recurrence and are now alive without evidence of disease after 62 and 28 months after the excision of the recurrent tumour. None of the 3 curetted cases showed recurrent tumour after a mean follow-up time of 16 years (10 to 18 years). No metastatic lesions have been observed.
*Conclusion:* In the majority of the cases CCC behaves as a very low-grade malignant tumour, however it is important to avoid underestimating a more aggressive lesion resembling a CCC; resection seems to be the better surgical approach.

**A17 Strategy of Treatment in Desmoid Tumours (Aggressive Fibromatosis in Children and Adults): to Avoid Absolutely Radiotherapy**

N. Delépine, S. Alkallaf, B. Markowsha, H. Cornille, I.M. Bigirimana, G. Delépine
*Avicenne Hospital, Bobigny, France*

In Desmoid tumour, frequent relapses are able to threaten life or conservation of limb. To point out the best indications of treatment, we reviewed our cases. From 1981 to 2000, we treated 50 patients (age 28 (1–70), locations were inferior limb (19), superior limb (17), axial (9), head and neck (5). 17 patients were seen at first hand, 33 with relapses. Treatment was adapted to each patient. En bloc extratumoural resection was performed each time, when surgery didn't expose to heavy functional consequences (22). 14 patients received chemotherapy. 10 received Interferon α, and 9 tamoxifen.
*Results:* Mean FU is 6 years ½>. 34 patients are in CR, 9 in SD, 6 PD, 1 died with thoracic complications. Following repeated surgery, functional consequences are numerous: 8 nerve palsies, 8 articular stiffness. Major functional consequences came from surgery with radiotherapy. In one, radiotherapy of a cervical lesion led to impossibility of surgery and to death. The analysis of our series leads to schematise the indications:

- If the tumour doesn't threaten life or limb, only surgery is needed as large as possible, avoiding functional consequences.
- When tumour relapses appear, Tamoxifen must be discussed.
- When the tumour invades noble structures, preoperative chemotherapy is useful, in order to soften the tumour and to help surgery.

*Conclusion:* in this unforeseeable illness, backgrounds of indications are respective risks of spontaneous evolution and of treatment. Besides surgery, needed in fast all cases, but often insufficient, it must be considered the value of interferon, tamoxifen and/or chemotherapy. The most important concept in the necessity to adapt a well-balanced treatment avoiding consequences and particularly radiotherapy. This last attitude leads to most heavy consequences and therapeutic deadlocks.

**A18 The Solitary Bone Lesion. A Consecutive Series of Aggressive Solitary Bone Lesions in Patients Over the Age of Forty**

J.T. Patton, J. Luscombe, S. Sommerville, R. Grimer
*Royal Orthopaedic Hospital, Birmingham, United Kingdom*

The purpose of this study is to investigate the causes and characteristics of the aggressive solitary bone lesion in patients over the age of forty. Over a four-year period, 318 patients over the age of forty were referred to our institution with what we would define as an aggressive solitary bone lesion. Further investigation and diagnostic biopsy as appropriate were performed in all patients. The lesions were then defined according to their radiological appearance, pathology and site. The nature of these lesions was then subdivided into several broad groups. A diagnosis of primary bone sarcoma was found in 30% of these lesions. Plasmacytoma, lymphoma and metastases accounted for 13% each. Benign bone tumours, infection and non-oncological diagnoses accounted for 9%, 6% and 16% of lesions respectively. Aggressive solitary bone lesions are often due to primary bone sarcomas. Metastases from a previously unrecognised primary malignancy account for less than one sixth of lesions. This study emphasises the need for appropriate investigation and biopsy of the aggressive solitary bone lesion.

**A19 Multiple Osteochondromas – Complications and Malignant Transformation**

Z. Matejovsky, Z. jr. Matejovsky, C. Povysil
*Hospital Bulovka, Prague 8, Czech Republic*

Multiple osteochondromas (MO), or multiple exostoses, diaphyseal aclasia, is a hereditary disorder with autosomal dominant heredity. We have reviewed 117 cases in our registration as to complications and malignant transformation.

*Methods & Results:* In the years 1966–1998 (33 years) we have registered 117 patients with MO, 64 men and 53 women. The youngest patient was a girl one-year of age, the daughter of one of our MO patients who died of malignant transformation. The eldest patient was 83 year, who was admitted for another diagnosis. He knew about his disorder since childhood, but he never did seek medical advice. The average age of patients at the time of diagnosis or when they were admitted for treatment was 20 year in men and 18 in women. The extent of skeletal involvement varied from a few small exostoses to severe deformities. Shortening of ulna and bowing of radius was frequent in severe cases and was treated by surgery. We registered two cases of femoral artery aneurysm caused by a needle sharp exostosis. In three of our patients chondrosarcomatous transformation occurred, two of them died, one is alive ten years after amputation. In addition, one large and increasing exostosis was removed from the pelvis, where only clinical signs of tumour progression were present and histology was unclear.

*Conclusion:* From our series of patients it seems that malignant transformation in MO is less frequent than usually given in the literature, amounting to 3, 4%. Nevertheless the patients should be under permanent control and should be informed about the possibility of progression into a malignant tumour.

**A20 Periosteal Osteosarcoma – Should They all have Chemotherapy? The Case for Another Emsos Study**

R.J. Grimer, M.P. Revell, N. Deshmukh
*Royal Orthopaedic Hospital, Birmingham, United Kingdom*

*Introduction:* Periosteal osteosarcomas are rare cartilage-rich bone tumours characterised by a juxtacortical eccentric position. While there is general agreement that wide surgical excision is required, there is a paucity of evidence regarding adjuvant therapy. Previous reports have not indicated any consistent approach to this to allow appraisal. We compare our experience with previous studies to evaluate both surgical and oncological methods of treating this tumour.

*Methods:* We retrospectively reviewed seventeen cases treated at our centre over sixteen years. All patients underwent surgery to remove the tumour. Limb reconstruction was usually necessary either at the time of primary excision or subsequently. Our policy was to use chemotherapy when the tumour showed any features of high grade.

*Results:* 10/17 patients received an endoprosthesis primarily, 4/17 had excision only and 3/17 excision and bone graft. 14/17 received chemotherapy. There was one local recurrence in the excision group treated by re-excision and endoprosthesis. To date, no deaths have resulted from recurrence or metastasis of the tumour although there have been two deaths from other causes.

*Discussion:* Our survival figures compare favourably with those reported previously. We suggest that this is probably the result of the evolution of surgical techniques and the contribution of adjuvant therapy. Further research is required to establish who benefits most from chemotherapy, but at present we would recommend it be considered in all cases of periosteal osteosarcoma showing any features of high grade.

**A21 Bone Giant Cell Tumour and Lung Metastases: A Surgical Approach**

M. Rocca, A. Briccoli, P. Picci, P Ruggieri, M Mercuri
*Istituti Ortopedici Rizzoli, Bologna, Italy*

Benign giant cell tumour of bone (GCT) rarely gives lung metastases. Data acquired from literature showed that lung metastases could follow different development pathways: spontaneous remission, remain unchanged for a long period, or increase. The Rizzoli Institute archives registered to December 2001 1090 patients with GCT (6% of all bone tumours). Sixteen patients (1.5%) underwent surgery, by the same team, for lung metastases. Nine were males, and seven females, the age ranging from 16 to 62. The site of primary bone tumour was the upper limb in 5 cases, lower limb in 10 cases, spine in 1 case. In 10 cases a local recurrence was evident. Lung metastases were unilateral in 8 cases. The average disease free interval from the onset of the primary tumour was 32 months in 14 patients; in 2 patients lung metastases were evident at presentation. All patients were operated with a contemporary mono or bilateral thoracotomy and a wedge resection was made. The average number of resected metastases was 6 (from 1 to 20). All patients were followed by repeated thoracic CT scan. Nine patients were freed by metastases and are continuous disease free (average follow-up 92.1 months). Five patients are alive with disease (average follow-up 55.8 months). Two patients died. The presence of a local recurrence is a risk factor for lung metastases, which can appear also after a long period of time following surgery of the primary tumour.

*Conclusion:* considering the low surgical risk and the opportunity for some patients to freed of the disease, resection of lung metastases from bone GCT is mandatory when their volume increases in time.

